# Supplementary material for: Causal Relationship between Aging and Anorexia Nervosa: A White-Matter-Microstructure-Mediated Mendelian Randomization Analysis
Source: Biomedicines. 2024 Aug 16;12(8):1874. doi: 10.3390/biomedicines12081874 (PMC11351342; doi:10.3390/biomedicines12081874)
Supplement: Supplementary file 1 [file biomedicines-12-01874-s001.zip › Supplementary Figure/Figure S8/Leaveoneout plot of MR analysis for TL to ubm-b-1989.pdf]

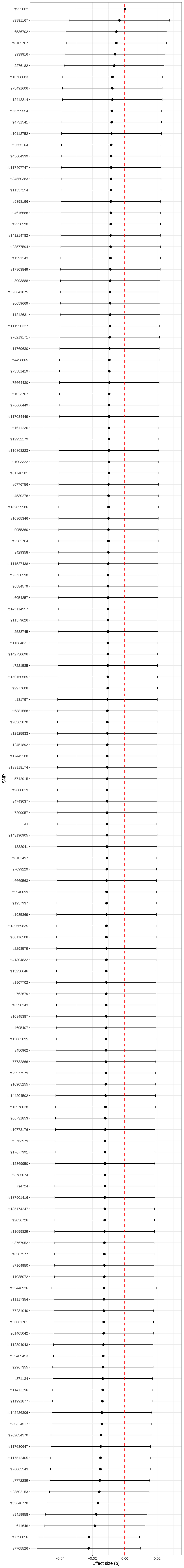
rs932002
rs3891167
rs6536702
rs8105767
rs939916
rs2276182
rs10768683
rs78491606
rs12412214
rs56799554
rs4731541
rs10112752
rs2555104
rs45604339
rs117407747
rs34550383
rs11557154
rs9398196
rs4616688
rs2230590
rs141214782
rs28577594
rs1291143
rs17803849
rs3093888
rs376641875
rs6659669
rs11212631
rs111950327
rs76219171
rs11769630
rs4498805
rs73581419
rs75664430
rs1023767
rs76666449
rs117034449
rs1611236
rs12932179
rs116863223
rs1003322
rs61748181
rs6776756
rs4530278
rs182059586
rs10805346
rs9955360
rs2282764
rs429358
rs111527438
rs73730598
rs6584579
rs6054257
rs145114957
rs11579626
rs2538745
rs11584821
rs142730696
rs7221585
rs150150565
rs2977608
rs131797
rs6881568
rs28363070
rs12925933
rs12451892
rs17445108
rs188918174
rs5742915
rs9600019
rs4743037
rs7209057
All
rs143190905
rs1332941
rs8102497
rs7099229
rs6669563
rs9940099
rs1957937
rs1985369
rs139669835
rs80116508
rs2293579
rs41304832
rs13230646
rs1907702
rs762679
rs6590343
rs10845387
rs4695407
rs13062095
rs450962
rs77732866
rs79977579
rs10905255
rs144204502
rs16978028
rs66731853
rs10773176
rs2763979
rs17677991
rs12369950
rs3785074
rs4724
rs137901416
rs185174247
rs2056726
rs11699829
rs3767952
rs6587577
rs7164950
rs11085072
rs35446936
rs11117354
rs77231040
rs56061761
rs61405042
rs112394943
rs59409453
rs2967355
rs871134
rs11412296
rs11991877
rs142426306
rs80324517
rs202034370
rs117630647
rs117512405
rs76065543
rs7772289
rs28502153
rs35640778
rs9419958
rs611646
rs7790856
rs7705526
SNP
−0.04
−0.02
0.00
0.02
Effect size (b)
